# Exosomes derived from apical papilla stem cells improve NASH by regulating fatty acid metabolism and reducing inflammation

**DOI:** 10.1186/s10020-024-00945-1

**Published:** 2024-10-26

**Authors:** Yifei Nie, Wenqing Meng, Duanqin Liu, Ziqing Yang, Wenhao Wang, Huiping Ren, Kai Mao, Weipeng Lan, Chuanhua Li, Zhifeng Wang, Jing Lan

**Affiliations:** 1 Department of Prosthodontics, School and Hospital of Stomatology, Cheeloo College of Medicine, Shandong University & Shandong Key Laboratory of Oral Tissue Regeneration & Shandong Engineering Research Center of Dental Materials and Oral Tissue Regeneration & Shandong Provincial Clinical Research Center for Oral Diseases, No. 44-1 Wenhua Road West, Jinan, 250012 Shandong China; 2 Department of Oral and Maxillofacial Surgery, School and Hospital of Stomatology, Cheeloo College of Medicine, Shandong University & Shandong Key Laboratory of Oral Tissue Regeneration & Shandong Engineering Research Center of Dental Materials and Oral Tissue Regeneration & Shandong Provincial Clinical Research Center for Oral Diseases, Jinan, Shandong China; 3 Department of Pediatric Dentistry, School and Hospital of Stomatology, Cheeloo College of Medicine, Shandong University & Shandong Key Laboratory of Oral Tissue Regeneration & Shandong Engineering Research Center of Dental Materials and Oral Tissue Regeneration & Shandong Provincial Clinical Research Center for Oral Diseases, No. 44-1 Wenhua Road West, Jinan, 250012 Shandong China

**Keywords:** Apical papilla stem cell-derived exosomes, NASH, AMPK, PPARα

## Abstract

**Background:**

Apical papilla stem cells (SCAPs) exhibit significant potential for tissue repair, characterized by their anti-inflammatory and pro-angiogenic properties. Exosomes derived from stem cells have emerged as safer alternatives that retain comparable physiological functions. This study explores the therapeutic potential of exosomes sourced from SCAPs in the treatment of non-alcoholic steatohepatitis (NASH).

**Methods:**

A NASH mouse model was established through the administration of a high-fat diet (HFD), and SCAPs were subsequently isolated for experimental purposes. A cell model of NASH was established in vitro by treating hepatocellular carcinoma cells with oleic acid (OA) and palmitic acid (PA). Exosomes were isolated via differential centrifugation. The mice were treated with exosomes injected into the tail vein, and the hepatocytes were incubated with exosomes in vitro. After the experiment, physiological and biochemical markers were analyzed to assess the effects of exosomes derived from SCAPs on the progression of NASH in both NASH mouse models and NASH cell models.

**Results:**

After exosomes treatment, the weight gain and liver damage induced by HFD were significantly reduced. Additionally, hepatic fat accumulation was markedly alleviated. Mechanistically, exosomes treatment promoted the expression of genes involved in hepatic fatty acid oxidation and transport, while simultaneously suppressing genes associated with fatty acid synthesis. Furthermore, the levels of serum inflammatory cytokines and the mRNA expression of inflammatory markers in liver tissue were significantly decreased. In vitro cell experiments produced similar results.

**Supplementary Information:**

The online version contains supplementary material available at 10.1186/s10020-024-00945-1.

## Background

Non-alcoholic fatty liver disease (NAFLD) is a chronic liver condition induced by metabolic imbalances within the body, characterized by abnormal lipid accumulation in hepatocytes (Zuo et al. 2024; Zhou et al. 2022a, b). Under persistent stimulation, it progresses from simple steatosis to inflammatory responses, fibrosis, and hepatocellular injury, eventually leading to end-stage liver cancer (Ferguson and Finck 2021; Paternostro and Trauner 2022; Friedman et al. 2018). NASH represents a more advanced manifestation of NAFLD. Metabolic disorders, such as type 2 diabetes, insulin resistance, and obesity, are intricately linked with NAFLD (Ferguson and Finck 2021). Currently, NAFLD remains the most prevalent chronic liver disease worldwide, significantly impacting children and young adults (Paternostro and Trauner 2022; Friedman et al. 2018). Furthermore, up to one-third of NASH patients may progress to end-stage liver disease, and NAFLD is a major contributor to the rising mortality rates associated with liver diseases. Presently, no pharmacological interventions for NAFLD have been approved, with lifestyle modifications being the primary management strategy (Sumida and Yoneda 2018; Zhong et al. 2024; Zhou et al. 2022a, b). However, the long-term efficacy of these interventions remains limited. Therefore, there is an urgent need to develop therapeutic agents to comprehensively address non-alcoholic fatty liver disease (Chalasani et al. 2018; Zobeiri et al. 2021).

Mesenchymal stem cell (MSCs)-derived exosomes are spherical vesicles approximately 100 nm in diameter, secreted by MSCs, and possess tissue repair functions similar to those of MSCs (Kalluri and LeBleu 2020; Zou et al. 2023;Zhao et al. 2023). Importantly, they circumvent issues typically associated with stem cell transplantation, such as tumorigenicity, high immunogenicity, and instability during in vivo delivery (Shi et al. 2022; Zhai et al. 2019). Consequently, this approach has evolved into a significant therapeutic strategy for treating liver diseases (Zuccarini et al. 2022; Xu et al. 2017). Numerous studies have demonstrated that exosomes secreted by MSCs from various tissues can ameliorate NASH (Wang et al. 2023a, b, c). For instance, exosomes derived from amniotic epithelial cells and human liver stem cells have shown inhibitory effects on liver inflammation and fibrosis in NASH mice (Goonetilleke et al. 2021; Bruno et al. 2020; Ohara et al. 2018). Similarly, exosomes from umbilical cord mesenchymal stem cells have been found to improve inflammation, abnormal lipid accumulation, and mitochondrial oxidative stress in NASH mice. Additionally, exosomes derived from bone marrow mesenchymal stem cells can enhance anti-apoptotic effects by promoting hepatic fatty acid oxidation, thereby ameliorating NASH in mice (Kang et al. 2022; Shi et al. 2022; El-Derany and AbdelHamid 2021). Apical papilla stem cells are obtained from incompletely developed human tooth root apical papilla tissue, often sourced from patients undergoing orthodontic tooth extraction (Zhai et al. 2019; Hilkens et al. 2017). Therefore, the potential of these readily accessible, cost-effective, and non-invasive stem cells in treating NASH remains to be explored.

Abnormal lipid metabolism in hepatocytes has been reported as a key factor in the pathogenesis of NASH (Diniz et al. 2021). AMP-activated protein kinase (AMPK) and peroxisome proliferator-activated receptor alpha (PPARα) have significant regulatory capabilities over lipid metabolism genes in hepatocytes (Smith et al. 2016; Herzig and Shaw 2018). Activation of AMPK and PPARα promotes the expression of fatty acid oxidation genes while reducing the expression of fatty acid synthesis genes, making it crucial for enhancing intracellular fatty acid β-oxidation (Sasaki et al. 2019; Garcia and Shaw 2017). Additionally, activation of PPARα can inhibit the phosphorylation of the pro-inflammatory transcription factor NF-kB (P65) (Montagner et al. 2016; Meeks et al. 2021), thereby exerting anti-inflammatory effects. Moreover, the polarization of macrophages is critical for the regulation of hepatic inflammatory cytokines (Zhang and Lang 2023; Guillot and Tacke 2019). Polarization of hepatic macrophages towards the M1 phenotype promotes the production of pro-inflammatory cytokines, whereas polarization towards the M2 phenotype promotes the production of anti-inflammatory cytokines (Sun and Matsukawa 2024; Barreby et al. 2022).

In this study, we established a NASH mouse model induced by HFD and a NASH cell model induced by OA and PA. The models were treated with exosomes to thoroughly investigate lipid metabolism and inflammatory phenotypes and mechanisms. The results demonstrated that exosomes can regulate lipid metabolism and inflammation in hepatocytes, providing new insights for the treatment of NASH.

## Methods

### Isolation and culture of SCAPs

With informed consent obtained from the patients, third molar extraction was performed on 12–16-year-old adolescents who had orthodontic extraction indications. Apical papilla tissues were isolated and rinsed with PBS buffer containing 5% penicillin/streptomycin (Gibco, USA) until the tissue appeared white. The tissues were then minced in 0.5 ml of 3 mg/ml type I collagenase (Solarbio, Beijing, China) solution to obtain a single-cell suspension. Subsequently, 0.5 ml of 3 mg/ml neutral protease (Solarbio, Beijing, China) solution was added. The mixture was incubated at 37 °C in a cell culture incubator with 5% CO₂ for 40 min, with inversion every 10 min. After incubation, the mixture was centrifuged at 10,000 g for 5 min, and the supernatant was discarded. The cell pellet was resuspended in the culture medium and cultured in flasks containing the culture medium, which consisted of α-MEM (Gibco, US) supplemented with 10% fetal bovine serum (Gibco, US) and 1% penicillin/streptomycin (Zhai et al. 2019).

This study was conducted in accordance with the protocol approved by the Ethics Committee of Biomedical Research Involving Human Subjects at the School of Stomatology, Shandong University (Approval Number: 20220809).

### Exosomes isolation

Apical papilla stem cells from passages 3–6 were cultured in the medium for 48 h, after which the culture medium was collected. Exosomes were extracted using a differential centrifugation method. The exosomes were separated in the following sequence, all performed at 4 °C: centrifugation at 300 g for 10 min, 2000 g for 10 min, 10,000 g for 30 min, and 100,000 g for 70 min. Cellular debris and large vesicles were removed at each step, leaving a translucent gel-like substance adhering to the tube wall, identified as exosomes. These were resuspended in PBS and subjected to a further 100,000 g centrifugation for 70 min to wash the exosomes (El-Derany and AbdelHamid 2021). Finally, the washed exosomes were resuspended in PBS, either used immediately or stored at −80 °C.

### Identification of exosomes

Morphological analysis of the isolated exosomes was conducted using transmission electron microscopy. The size distribution of the exosomes was determined through nanoparticle tracking analysis (NTA). Furthermore, the presence of exosomal surface markers, including TSG101, Calnexin, HSP70, and CD63, was confirmed via immunoblotting.

### Cell culture and treatment

The cell culture medium consisted of DMEM (Gibco, US), 10% FBS (Gibco, US), and 1% penicillin–streptomycin (Gibco, US). Human hepatocellular carcinoma cells HepG2 (Procell, Wuhan, China) was cultured in the aforementioned medium in a 37 °C incubator with 5% CO_2_. The cells were passaged every 2–3 days. Cells were treated for 24 h with 0.5 mM OA, 0.25 mM PA (Kunchuang, China), and exosomes derived from apical papilla stem cells (100 μg/ml). The specific treatment protocols are presented in Table 1.

**Table 1:** Cell treatment

Group	OA/PA (mM)	Exo co-incubation (μg/ml)	Time (h)
Control	–	–	24
OA/PA	0.5/0.25	–	24
EXO-100 μg/ml	0.5/0.25	100	24

### Identification of adipogenic, osteogenic and chondrogenic differentiation of MSCs

SCAPs were seeded in 24-well plates at a density of 5 × 10^4^ cells/well and cultured in a 37 °C, 5% CO_2_ incubator, with passages conducted every 2–3 days. After 2–3 days of culture in the standard medium, the cells were replaced with either adipogenic or osteogenic induction medium. After 21 days of induction, the cells were stained with Oil Red O (Solarbio, Beijing, China) or Alizarin Red S (Solarbio, Beijing, China) and photographed under a microscope. 2.5 × 10^5^ cells were placed in a 15 ml centrifuge tube, and 0.5 ml of chondrogenic induction complete medium (Procell, Wuhan, China) was added. The tube was then centrifuged at 150 g for 5 min. After centrifugation, the cap of the tube was gently loosened, and the tube was incubated at 37 °C with 5% carbon dioxide in a cell culture incubator. The chondrogenic induction complete medium was replaced every 2–3 days. After 28 days, the chondrospheres were fixed with neutral formaldehyde, embedded in paraffin, and then sectioned. Alcian Blue staining was subsequently performed on the sections.

### Flow cytometric identification of hepatocyte surface marker proteins

Apical papilla stem cells were prepared into a cell suspension in cell culture medium. A 100 μL aliquot of the cell suspension was incubated with specific antibodies at 4 °C in the dark for 30 min. The cells were then washed twice with cold PBS and analyzed by flow cytometry. The specified primary and secondary antibodies are listed in Table 3.

### Animal treatment

Male C57BL/6 J mice (5–6 weeks old, 17–20 g) were purchased from Jiangsu Xietong Pharmaceutical Bio-engineering Company (Jiangsu, China). The mice were randomly divided into four groups, each containing six mice. After a 1-week acclimation period, the mice were fed HFD (40% kcal fat, 20% kcal fructose, 2% kcal cholesterol) to establish a NASH mouse model. All mice were housed in open cages at a temperature of 25 °C with a 12-h light/dark cycle. The first group was fed a regular diet. The second group was fed an HFD for 18 weeks and received tail vein injections of PBS for the last 6 weeks. The third group was fed an HFD for 18 weeks and received tail vein injections of apical papilla stem cell-derived exosomes (50 μg per mouse) for the last 6 weeks. The fourth group was fed an HFD for 18 weeks and received tail vein injections of apical papilla stem cell-derived exosomes (100 μg per mouse) for the last 6 weeks. Injections were administered twice weekly (Kang et al. 2022). During the 6 weeks of exosomes treatment, we recorded body weights seven times. At the end of the eighteenth week, the animals were anesthetized with an intraperitoneal injection of 1% sodium pentobarbital. Blood was collected from the retro-orbital sinus for serum separation, and the mice were then sacrificed for liver tissue collection and weighing. Liver tissue samples were collected for physiological and biochemical analyses. The specific treatment protocols are presented in Table 2.

**Table 2:** Animal treatment

Group	Number of mice	Diet	Injections	Injection frequency
Control	6	Regular diet	–	–
HFD	6	HFD for 18 weeks	PBS for last 6 weeks	Twice weekly
Exo-50 μg/mouse	6	HFD for 18 weeks	Exo (50 μg) for last 6 weeks	Twice weekly
Exo-100 μg/mouse	6	HFD for 18 weeks	Exo (100 μg) for last 6 weeks	Twice weekly

This study was conducted in accordance with protocols approved by the Animal Ethics Committee of the Stomatology School at Shandong University (Approval No. 20220801).

### Histological analysis of liver

Mouse liver tissues were preserved in 4% paraformaldehyde. Portions of the tissue were subjected to graded ethanol dehydration and xylene clearing, followed by paraffin embedding and sectioning into 2–4 μm thick paraffin sections. These sections were stained with hematoxylin and eosin (Solarbio, Beijing, China). Other liver tissues were embedded in OCT (Wuhan, Cervell, China) compound, frozen, sectioned into 7 μm thick slices, and stored at −20 °C. These frozen sections were stained with Oil Red O and counterstained with hematoxylin.

### Assessment of hepatotoxicity, hepatic triglyceride content and serum inflammatory cytokine levels

The levels of aspartate aminotransferase (AST) and alanine aminotransferase (ALT) in serum reflect the extent of hepatotoxicity. Their concentrations were measured according to the instructions provided with the ALT/AST assay kit (Solarbio, Beijing, China). The hepatic triglyceride (TG) content was determined following the protocol specified in the TG assay kit (Solarbio, Beijing, China). The levels of tumor necrosis factor-α (TNF-α) and interleukin-6 (IL-6) in serum were measured using enzyme-linked immunosorbent assay (ELISA) kits from 4A Biotech (Beijing, China), following the manufacturer’s instructions.

### Nile Red and Oil Red O staining assays for cells

After treating HepG2 cells for 24 h using the method described in “Cell culture and treatment” section, the cells were fixed with 4% paraformaldehyde at room temperature for 10 min. They were then incubated with Nile Red (Solarbio, Beijing, China) staining solution for 10 min in the dark, followed by DAPI (Beyotime, Beijing, China) staining for the nuclei. Similarly, after fixing the cells with 4% paraformaldehyde at room temperature for 10 min, the cells were stained with Oil Red O. Finally, the cells were observed under a microscope.

### Detection of cellular ROS

Seed cells at a density of 1 × 10^5^ cells/well in a 6-well plate. After treatment as described in step 2.4, remove the culture medium and add the ROS fluorescent probe solution (Beyotime, Beijing, China). Incubate at 37 °C for 30 min, then observe under a fluorescence microscope.

### Western blotting

Proteins were extracted from liver tissues, exosomes, and hepatocytes by lysing with RIPA buffer (Solarbio, Beijing, China). The total protein concentration was measured using a BCA protein assay kit (Beyotime, Beijing, China). The lysates were separated using a 12% sodium dodecyl sulfate–polyacrylamide gel (SDS-PAGE) (Beyotime, Beijing, China). After electrophoresis, the proteins in the gel were transferred onto polyvinylidene difluoride (PVDF) membranes (Millipore Corp, Billerica, MA, USA). The membranes were then blocked with 5% bovine serum albumin for 1 h. Following blocking, the membranes were incubated overnight at 4 °C with primary antibodies against SREBP-1c, AMPK, p-AMPK, PPAR-α, CPT-1α, NF-κB, P-NF-κB, TSG101, CD63, CD81, Calnexin, and β-actin. Finally, the membranes were incubated with anti-rabbit IgG secondary antibodies for 1 h at room temperature. Protein levels were detected using the ECL Plus detection system (Thermo Fisher Scientific). The specified primary and secondary antibodies are listed in Table 3.

**Table 3:** Antibodies used for western blotting

Antibodies		
Antibodies	Dilution	Description
Anti-AMPK	1:1000	Cell Signaling Technology, 5831T
Anti-CPT1α	1:1000	Immunoway, YN3388
Anti-NF-KB	1:1000	Cell Signaling Technology, 8242T
Anti-p-AMPK	1:1000	Cell Signaling Technology, 2535T
Anti-p-NF-KB	1:1000	Cell Signaling Technology, 3033T
Anti-PPARα	1:1000	Immunoway, YT3835
Anti-SREBP1c	1:1000	Immunoway, YT6055
Anti-β-actin	1:2000	BIOSS, bs-0061R
Calnexin	1:1000	Abcam, ab22595
CD63	1:1000	Abcam, ab134045
HRP-labeled antibody	1:10,000	Immunoway, RS0002
HSP70	1:1000	Abcam, ab2787
TSG101	1:1000	Abcam, ab1225011
CD11	1:1000	ThermoFisher, CD11B01
CD44	1:1000	ThermoFisher, 11-0441-82
CD45	1:1000	ThermoFisher, 11-0451-82
CD90	1:1000	ThermoFisher, 11-0909-42

### Quantitative real-time polymerase chain reaction (qPCR) measurements

Using TRIZOL reagent, total RNA was extracted from mouse liver tissues and HepG2 cells. The reverse transcription kit (Vazyme, Nanjing, China) was employed to synthesize single-stranded DNA. Real-time quantitative PCR (qPCR) analysis was performed using SYBR Green PCR Master Mix (Vazyme, Nanjing, China) following the established methods. Glutamate dehydrogenase served as the reference gene, and the primers were purchased from BoShang Biotechnology Co, Ltd. (China). The primer sequences are listed in Table 4.

**Table 4:** Primer sequences for qPCR

Primer sequences used for real-time PCR		
Gene	Forward	Reverse
ACACA (mouse)	GGGAACATCCCCACGCTAAA	GAAAGAGACCATTCCGCCCA
ACOX (mouse)	GGAACATCATCACAGGGGCT	CAGAGCCAAGGGTCACATCC
Arg-1 (mouse)	TGTCCCTAATGACAGCTCCTT	GCATCCACCCAAATGACACAT
CD206 (mouse)	ACGAGCAGGTGCAGTTTACA	ACATCCCATAAGCCACCTGC
CPT1α (mouse)	AAGAACATCGTGAGTGGCGT	ACCTTGACCATAGCCATCCAG
FABp5 (mouse)	GGGAAGGAGAGCACGATAACA	TGCACCTTCTCATAGACCCGA
FASN (mouse)	GGCCCCTCTGTTAATTGGCT	GGATCTCAGGGTTGGGGTTG
GAPDH (mouse)	TGTCTCCTGCGACTTCAACA	GGTGGTCCAGGGTTTCTTACT
IL10 (mouse)	GCTCTTGCACTACCAAAGCC	CTGCTGATCCTCATGCCAGT
IL-1β (mouse)	GTGTCTTTCCCGTGGACCTT	AATGGGAACGTCACACACCA
IL-6 (mouse)	CTTCTTGGGACTGATGCTGGT	CTCTGTGAAGTCTCCTCTCCG
PPARα (mouse)	CTGGGCAAGAGAATCCACGA	AAGCGTCTTCTCGGCCATAC
SREBP-1c (mouse)	GGCCCGGGAAGTCACTGT	GGAGCCATGGATTGCACATT
TNF-α (mouse)	CGGGCAGGTCTACTTTGGAG	ACCCTGAGCCATAATCCCCT
PPARα (human)	GCTTCGCAAACTTGGACCTG	ACAGAAGACAGCATGGCGAA
SREBP-1c (human)	CCATGGATTGCACTTTCGAA	CCAGCATAGGGTGGGTCAAA
CPT1α (human)	TCACCGCAGGAGACAGAGTT	CCACCTGTCGTAACATCGGC
FASN (human)	CTCAGCCGCCATCTACAACA	GCCAGCGTCTTCCACACTAT
GAPDH (human)	GCACCGTCAAGGCTGAGAAC	TGGTGAAGACGCCAGTGGA

### Statistical analysis

The statistical analysis was conducted using Prism software (GraphPad 10.1.0) to meet high academic research standards. Data are expressed as mean ± standard error of the mean (SEM). Comparisons between the experimental group and the control group were performed using independent samples *t* test or one-way analysis of variance (ANOVA). A *p* value of less than 0.05 was regarded as statistically significant.

## Results

### Characterization of SCAPS-MSCs and exosomes

**Fig. 1:**
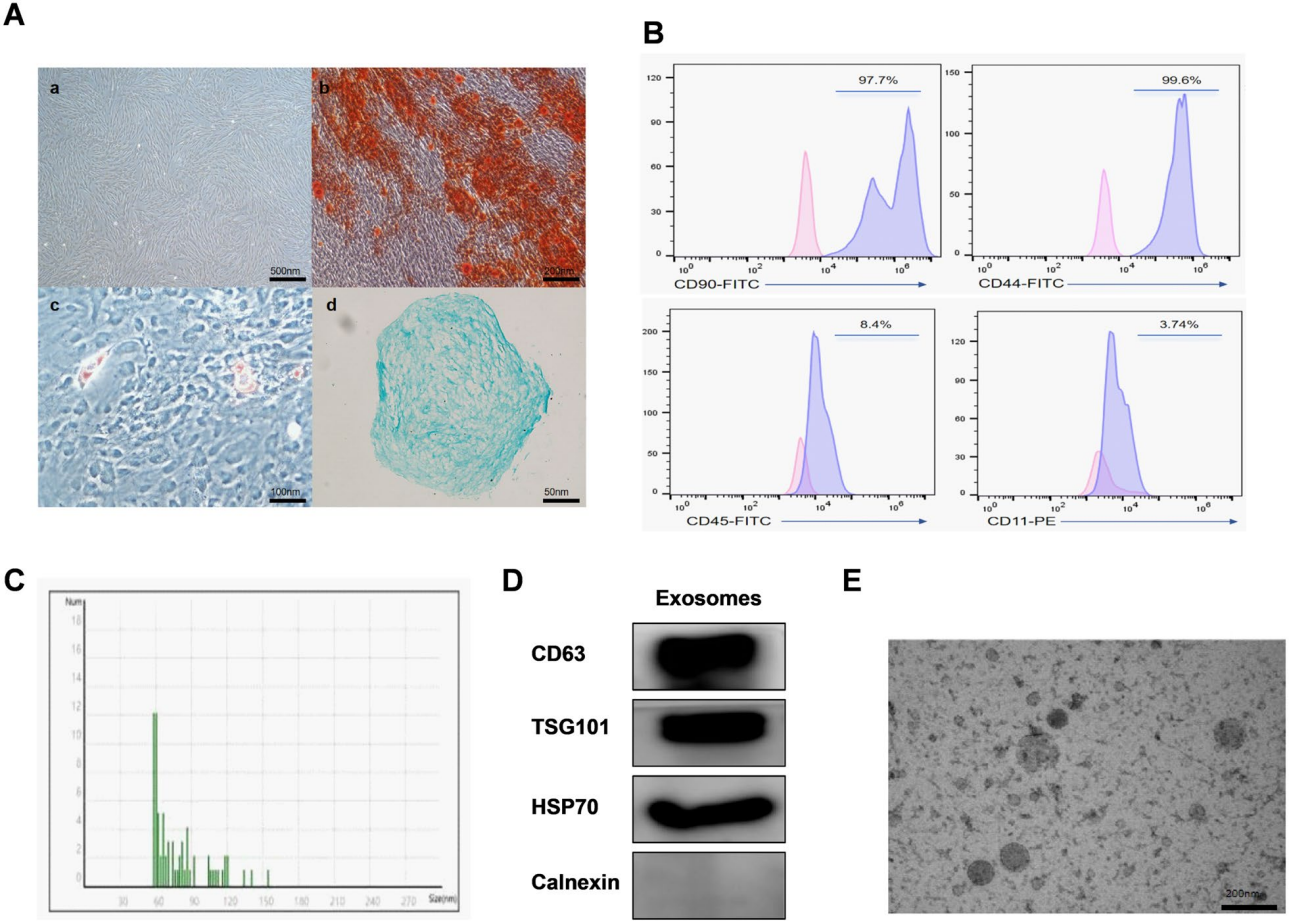
Characterization of SCAPS-MSCs and exosomes. **A** (a) Under light microscopy, third-generation apical papilla stem cells were observed; (b, c, d) Osteogenic, adipogenic and Chondrogenic differentiation of SCAPs. **B** Flow cytometry was used to detect the surface proteins of exosomes: CD11, CD45, CD44, and CD90. **C** The size distribution of exosomes derived from SCAPs was illustrated using nanoparticle tracking analysis (NTA). **D** The expression of TSG101, CD63, HSP70, and Calnexin in exosomes derived from SCAPs was revealed by immunoblot analysis. **E** Images from transmission electron microscopy depict the structural characteristics of exosomes derived from SCAPs, with a scale bar of 200 nm

The morphology of third-generation apical papilla stem cells, captured by optical electron microscopy, appears uniformly spindle-shaped. Trilineage differentiation assays validated the stemness of these cells, demonstrating their potential to differentiate into adipocytes, osteocytes, and chondrocytes (Fig. 1A). The identification of SCAPs was performed using flow cytometry. As shown in the Fig. 1B, the majority of SCAPs highly express CD90 and CD44, whereas cells expressing CD45 and CD11 are rare. Nanoparticle Tracking Analysis (NTA) was used to measure the diameter of the isolated exosomes, revealing an average diameter of 82 nm (Fig. 1C). Western blot analysis showed significant expression of the exosomal surface marker proteins TS101, CD63, and HSC70, whereas the endoplasmic reticulum-specific molecule Calnexin was negatively expressed (Fig. 1D). Transmission electron microscopy examination of the exosome morphology revealed them to be biconcave, disc-shaped structures (Fig. 1E).

### Apical papilla stem cell exosomes treatment alleviates systemic and hepatic damage induced by HFD in mice

**Fig. 2:**
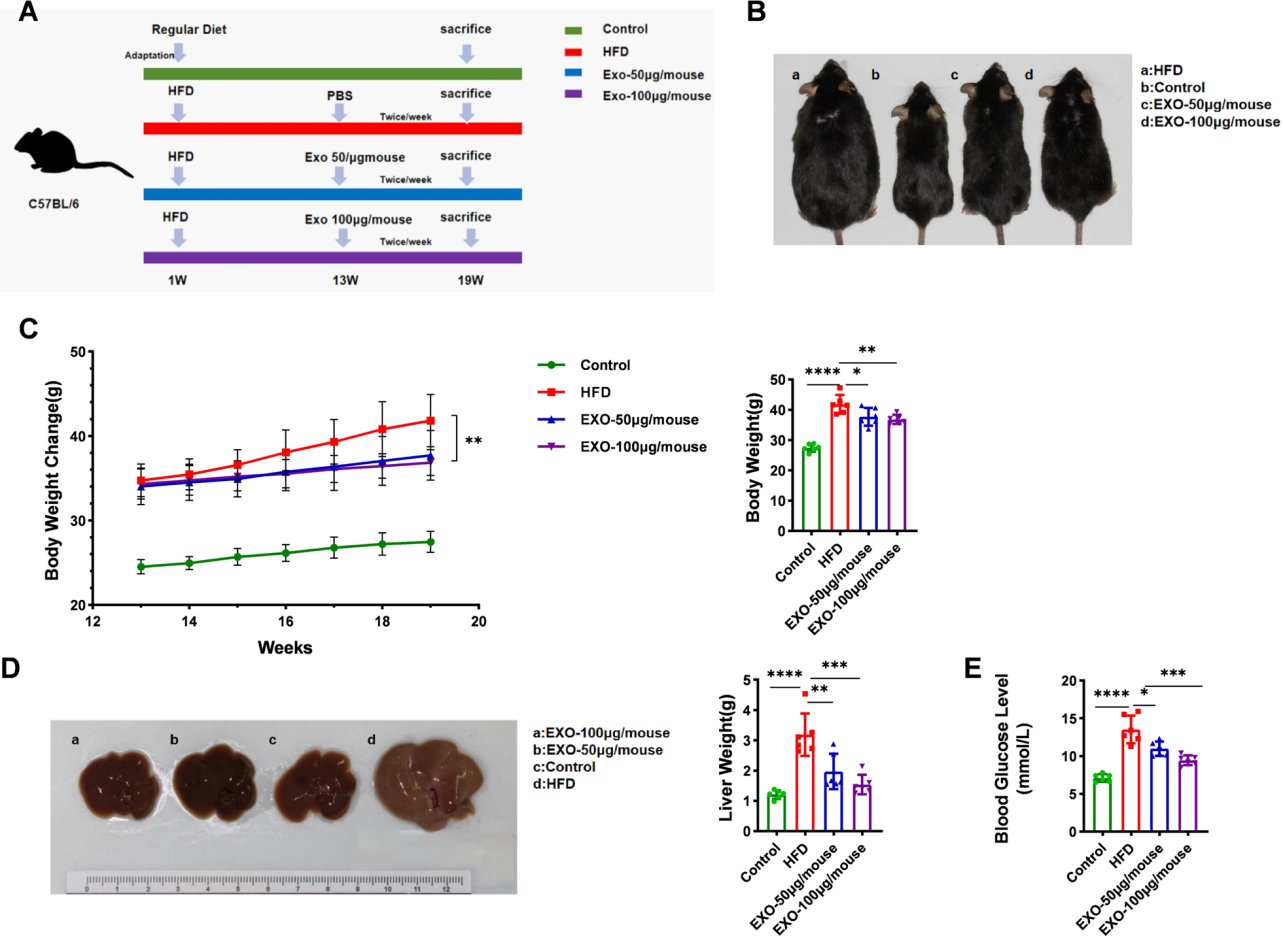
Exosomes derived from SCAPs alleviates systemic and hepatic damage. **A** The treatment protocols for the four groups of mice. **B** Photographs representing the final body size of mice in each group. **C** Over the 7-week period, changes in body weight and the final body weight of mice in each group (*n* = 6 per group). **D** Photographs representing liver size in mice from each group and the liver weights of mice in each group (*n* = 6 per group). **E** In each group of mice (*n* = 6), blood glucose levels are presented. Data are presented as Mean ± SD, with * *p* < 0.05, ** *p* < 0.01, and *** *p* < 0.001

Figure 2A shows the treatment of the four groups of mice. After 12 weeks of HFD, the body weights of the three groups of mice induced by the HFD were relatively consistent, and compared to the normal diet group, the HFD-induced mice showed a more rapid weight gain. Following 6 weeks of exosome treatment, the exosome-treated mice exhibited a significantly slower weight gain trend compared to the HFD group. Additionally, the final body weight of the exosomes-treated mice was lower than that of the mice on the HFD alone (Fig. 2C). At the end of week 19, body comparisons through photographs revealed that the HFD group mice were noticeably obese, whereas the exosomes treatment was able to mitigate this obesity (Fig. 2B). These results suggest that exosomes intervention significantly improved HFD-induced obesity in mice. Complete livers were isolated from the mice, and comparative photographs and weight measurements of the livers from each group were taken. The results showed that compared to the normal diet group, the HFD led to enlarged, pale livers in mice, while exosomes intervention reversed this condition (Fig. 2D). The experiment also measured the final blood glucose levels of the mice. It was found that, compared to the regular diet group, the blood glucose levels were significantly higher in the high-fat diet group. However, this increase was reversed following exosomes intervention (Fig. 2E). Furthermore, the experimental results demonstrated that a treatment dose of 100 μg per mouse was more effective than 50 μg per mouse.

### SCAPS exosomes alleviate hepatic lipid accumulation induced by HFD in mice

**Fig. 3:**
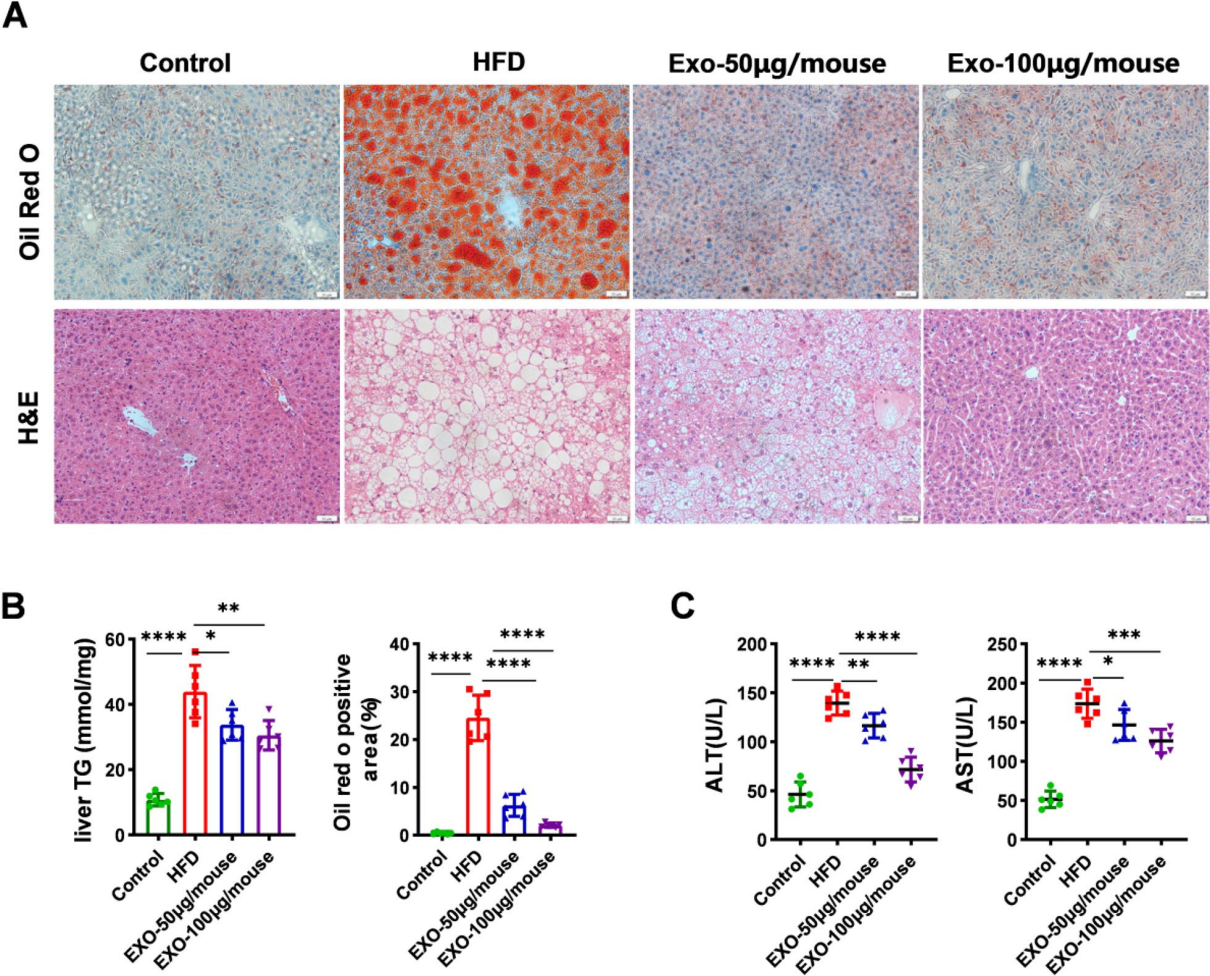
SCAPS exosomes alleviate hepatic lipid accumulation. **A** Histopathological images of representative liver sections: HE staining and oil red O staining (magnification ×20, scale bar = 50 μm). **B** The Oil Red O positive area and hepatic TG content in different groups of mice (*n* = 6). **C** In each group of mice (*n* = 6), plasma alanine aminotransferase (ALT) and aspartate aminotransferase (AST) levels. Data are presented as mean ± SD, * *p* < 0.05, ** *p* < 0.01, *** *p* < 0.001

Histological staining was performed on the livers of each group of mice. Hematoxylin and eosin (HE) staining results showed significant hepatocyte swelling and vacuolar degeneration in the livers of the HFD group mice (Fig. 3A). Oil Red O staining and area assessment also indicated substantial fat accumulation in the livers of the HFD group mice (Fig. 3A). However, exosomes intervention reversed these histological symptoms and reduced hepatic lipid accumulation (Fig. 3A). The measurement of hepatic TG content and Oil Red O staining area yielded results consistent with the aforementioned findings (Fig. 3B). Serum aspartate aminotransferase (AST) and alanine aminotransferase (ALT) levels were measured to assess liver damage in each group (Fig. 3C). The results demonstrated that exosome intervention could reverse the liver damage induced by the HFD in mice.

### SCAPs exosomes regulate liver lipid metabolism in HFD-induced NASH in mice

**Fig. 4:**
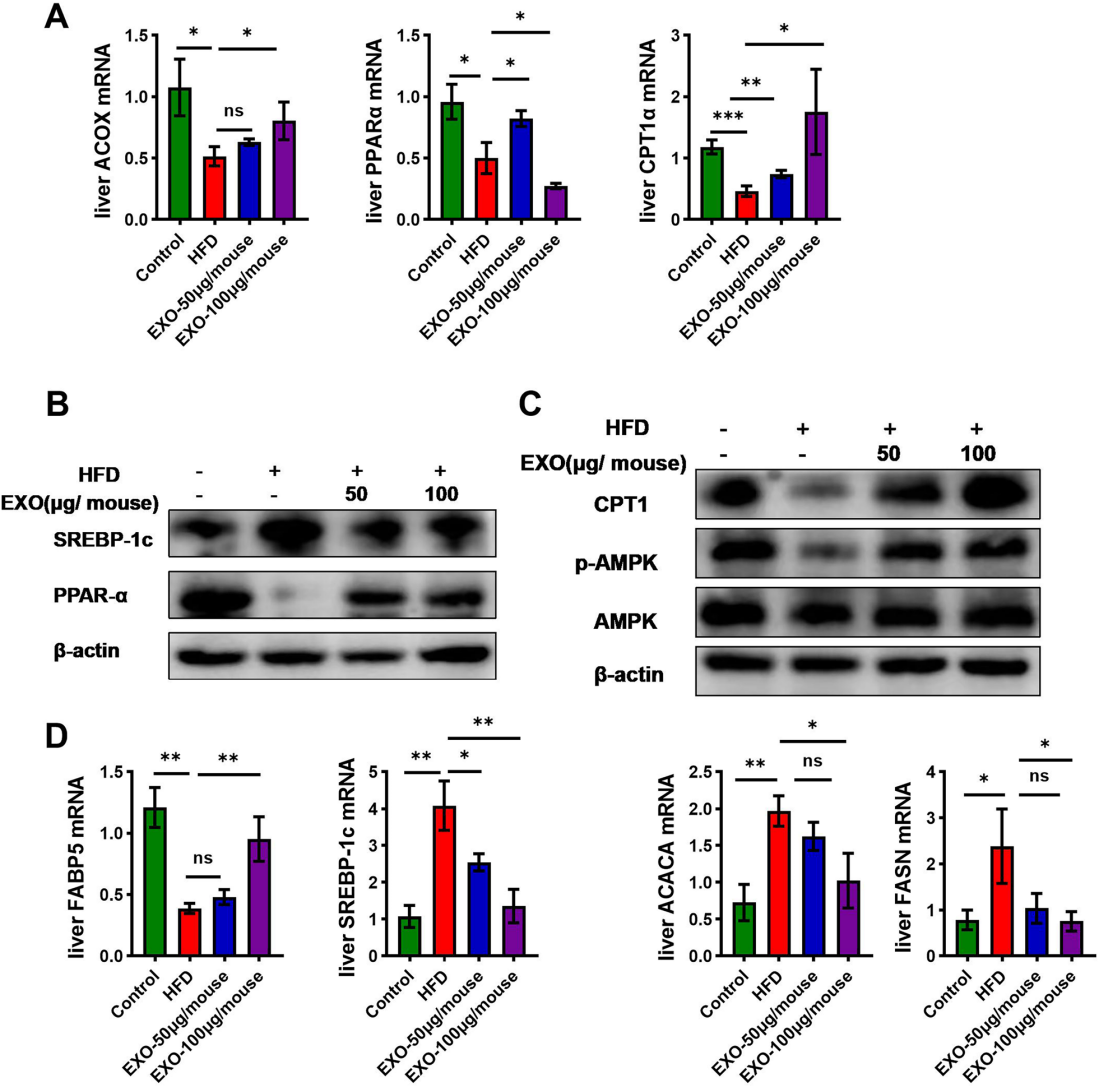
SCAPs exosomes regulate liver lipid metabolism. **A** The mRNA levels of PPARα, ACOX, and CPT1 were analyzed by quantitative polymerase chain reaction (qPCR) in liver tissues of mice across different groups (*n* = 3). **B** The protein expression of hepatic PPARα and SREBP1c was assessed via immunoblotting in liver tissues of mice across different groups. **C** The protein expression of CPT1α, AMPK and phosphorylated AMPK (p-AMPK) proteins was assessed via immunoblotting in liver tissues of mice across different groups. **D** The mRNA levels of FABP5, SREBP1c, FASN and ACACA were analyzed by quantitative polymerase chain reaction (qPCR) in liver tissues of mice across different groups (*n* = 3). Data are presented as mean ± SD, * *p* < 0.05, ** *p* < 0.01, *** *p* < 0.001

The phosphorylation activation of AMPK leads to the inactivation of the downstream gene ACC and simultaneously promotes the expression of CPT1α, thereby enhancing the process of fatty acid oxidation. PPARα regulates its downstream genes involved in fatty acid oxidation, storage, transport, and synthesis, affecting liver lipid metabolism. The study investigated the expression of these key hepatic lipid metabolism genes and found that the mRNA expression levels of fatty acid oxidation-related genes such as PPARα, Acox, and CPT1α, and fatty acid transport-related gene FABP5, were significantly reduced after HFD induction (Fig. 4A, D), while the mRNA expression levels of lipid synthesis genes SREBP1c, FASN, and ACC were significantly increased (Fig. 4D). However, exosomes intervention reversed these changes. Western blot results similarly showed that exosomes intervention promoted the phosphorylation of AMPK protein, increased the protein expression of hepatic PPARα and CPT1α, and decreased the expression of SREBP1c (Fig. 4B, C). All these results exhibited significant concentration dependency.

### Exosomes derived from SCAPs alleviated liver inflammation in mice, potentially associated with M2 macrophage polarization

**Fig. 5:**
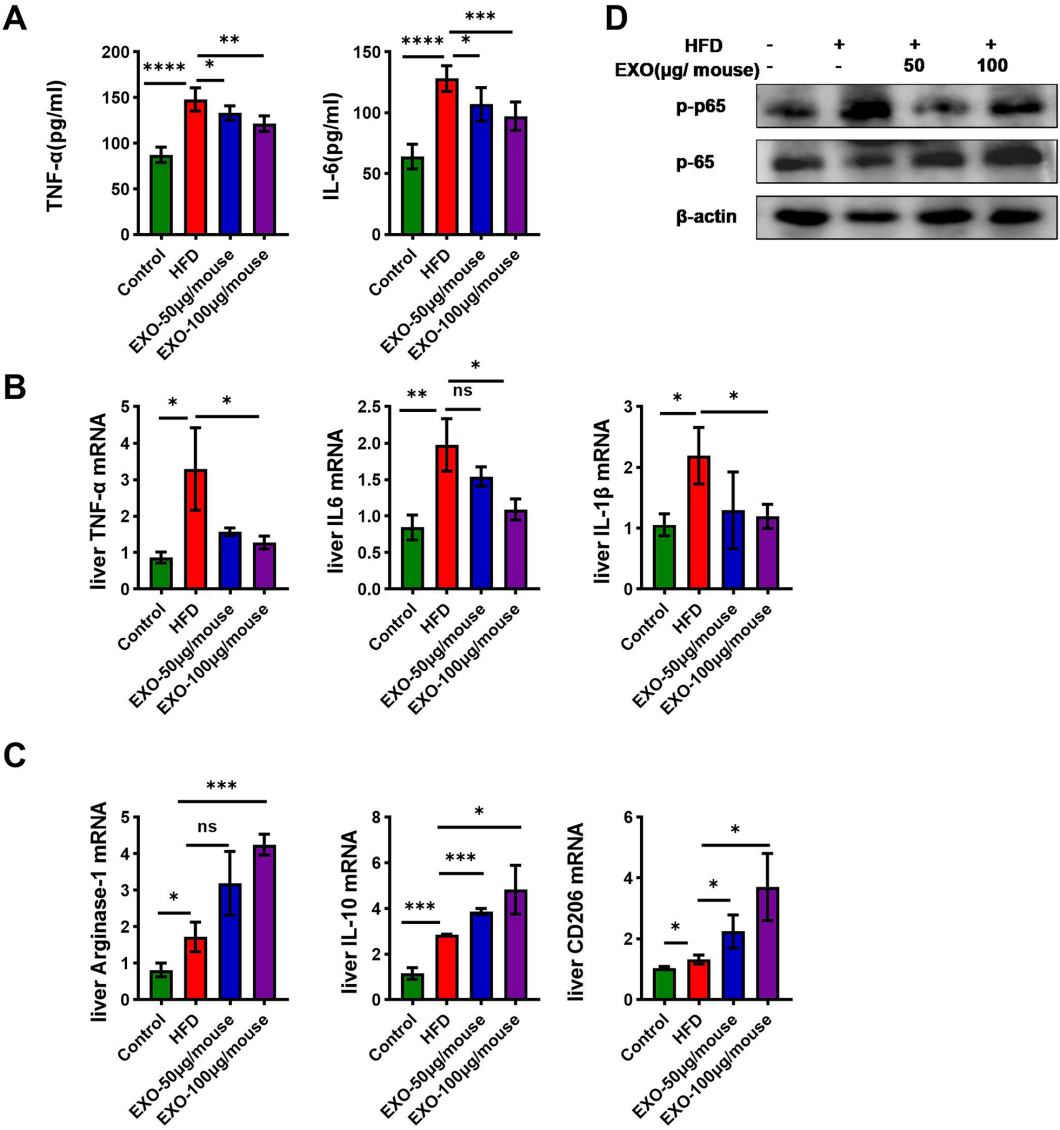
SCAPS exosomes alleviated liver inflammation in mice. **A** Serum concentrations of tumor necrosis factor-α and interleukin-6 were determined using the ELISA method (*n* = 6). **B** The mRNA levels of inflammatory cytokines in liver tissues were quantified using quantitative polymerase chain reaction (qPCR) (*n* = 3). **C** The mRNA levels of CD206, IL10 and Arg1 were assessed through qPCR in liver tissues of mice (*n* = 3). **D** Immunoblotting analysis showing phosphorylated nuclear factor-kB (p-p65) and total nuclear factor-kB (P65) in liver tissues of mice (*n* = 3). Data are presented as mean ± SD, * *p* < 0.05, ** *p* < 0.01, *** *p* < 0.001

This study measured the levels of inflammatory factors in the serum of mice and their mRNA expression levels. The results showed that, compared to the regular diet group, serum levels of tumor necrosis factorα (TNFα) and IL6 were significantly elevated in mice following HFD induction. Additionally, the expression levels of TNFα, IL6 and IL1β mRNA in the liver were also significantly increased. Intervention with exosomes reversed these changes (Fig. 5A, B). NF-kB, upon activation, can regulate the expression of various pro-inflammatory genes such as TNFα and IL6. Consistent with these findings, western blot results showed that the phosphorylation levels of NF-kB in the liver of mice were significantly elevated following HFD feeding, while exosomes intervention reversed this change (Fig. 5D). M2 macrophages represent a functional state of macrophages capable of secreting anti-inflammatory factors and inhibiting the production of pro-inflammatory factors, thereby mitigating the inflammatory response. Compared to the HFD group, exosomes intervention resulted in significantly increased expression levels of the M2 macrophage surface marker CD206, the anti-inflammatory marker arginase-1, and the anti-inflammatory cytokine IL10 mRNA in the liver of mice (Fig. 5C). These results suggest that exosomes intervention may promote the polarization of macrophages towards the M2 phenotype.

### SCAPS exosomes improve NASH in vitro

**Fig. 6:**
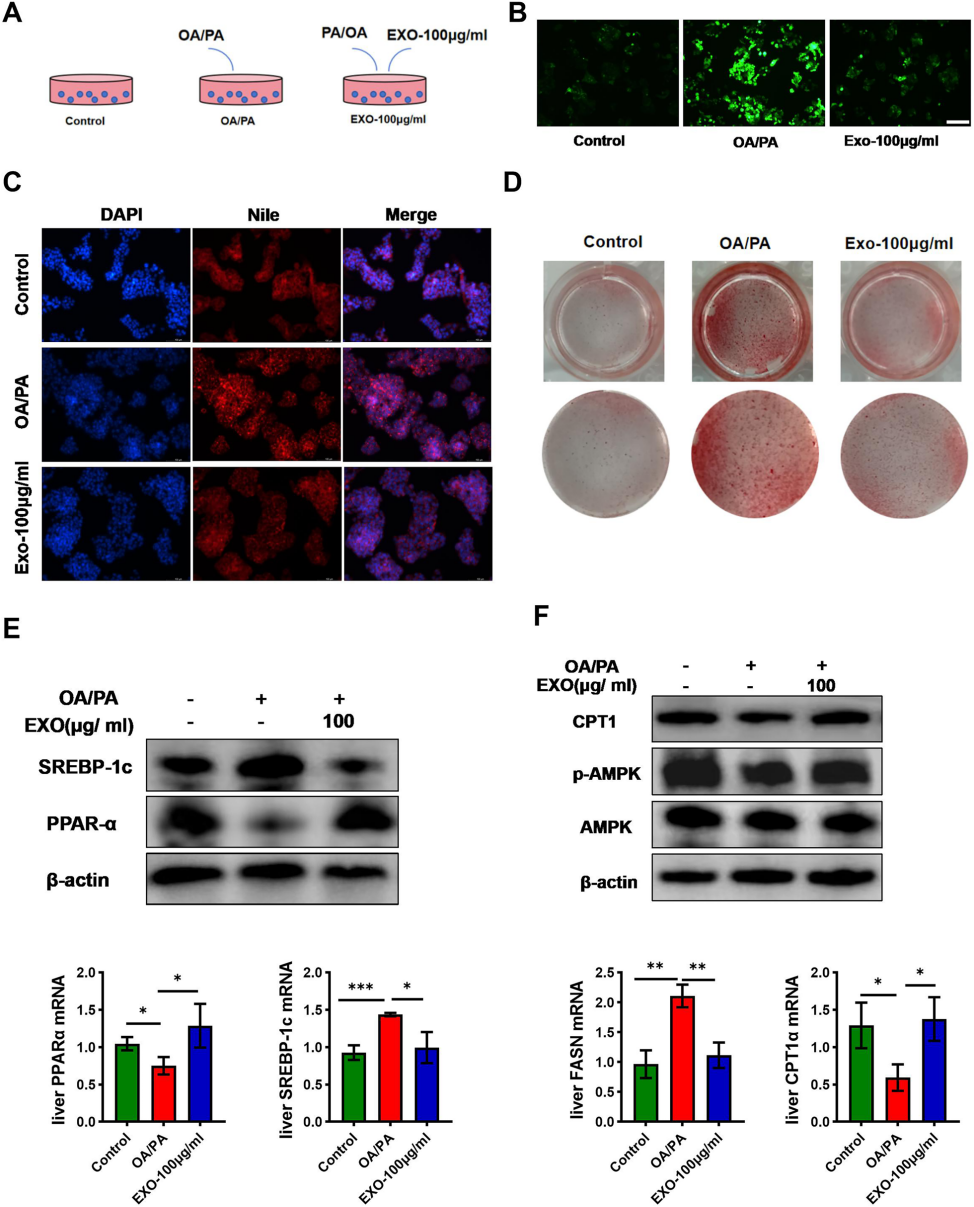
SCAPS exosomes improve NASH in vitro. **A** The treatment protocols for the three groups of cells. **B** Fluorescence intensity of cells in each group after ROS fluorescence staining. **C** Fluorescence intensity of cells in each group after Nile Red and DAPI staining. **D** Staining intensity of cells in each group after Oil Red staining. **E** The mRNA levels of PPARα and SREBP1c in liver tissues were quantified using quantitative polymerase chain reaction (qPCR) (*n* = 3). The protein expression of PPARα and SREBP1c was assessed via immunoblotting in liver tissues of mice across different groups. **F** The mRNA levels of CPT1α and FASN in liver tissues were quantified using quantitative polymerase chain reaction (qPCR) (*n* = 3). The protein expression of CPT1α, AMPK and phosphorylated AMPK (p-AMPK) proteins was assessed via immunoblotting in liver tissues of mice across different groups. Data are presented as mean ± SD, * *p* < 0.05, ** *p* < 0.01, *** *p* < 0.001

To further verify the ameliorative effect of exosomes on NASH, we conducted in vitro cell experiments. As shown in Fig. 6A, the cells were divided into three groups: the first group was normally induced with regular culture medium, the second group was induced with OA/PA to construct a NASH model in HepG2 cells for 24 h, and the third group was co-incubated with exosomes on the basis of OA/PA induction. After the experiment, the cells were stained with Oil Red O and Nile Red. The results showed that the cells treated with OA and PA exhibited increased Nile Red fluorescence intensity and Oil Red O area, while this phenomenon was significantly improved after co-incubation with exosomes (Fig. 6C, D). The intracellular reactive oxygen species (ROS) content can reflect the degree of cell damage to some extent. ROS fluorescent probe staining indicated that the ROS fluorescence intensity of cells significantly increased after OA/PA induction, and co-incubation with exosomes could reverse this phenomenon (Fig. 6B). Finally, the expression levels of hepatic lipid metabolism genes in the cells were detected. Exosomes promoted the mRNA expression levels of fatty acid oxidation genes PPARα and CPT1α, and decreased the mRNA expression levels of lipid synthesis genes SREBP1c and FASN (Fig. 6E, F). Western blot results showed that the phosphorylation level of AMPK protein in the cells increased after co-incubation with exosomes, and the protein expression of hepatic PPARα and CPT1α significantly increased, while the protein expression of SREBP1c decreased (Fig. 6E, F). These results were consistent with the in vivo experiments.

## Discussion

NAFLD is a chronic liver condition characterized by the accumulation of fat in the liver in the absence of significant alcohol consumption (Simon et al. 2021; Younossi et al. 2018). NASH is a severe form of NAFLD, often closely associated with metabolic syndrome (Gjorgjieva et al. 2019). The pathological progression of NASH primarily includes the following stages: hepatic steatosis, oxidative stress and inflammation, hepatocyte injury and inflammatory response, liver fibrosis, ultimately progressing to cirrhosis and hepatocellular carcinoma (Zhou et al. 2022a, b; Ferguson and Finck 2021). It has been reported that the global prevalence of NAFLD has now reached 25%, with the incidence of NAFLD increasing annually worldwide, particularly high in Western countries. In Asian countries, the incidence of NASH is also rapidly rising due to the westernization of lifestyle and increasing rates of obesity (Simon et al. 2021; López et al. 2023). Currently, the treatment strategies for NASH primarily focus on lifestyle interventions and pharmacotherapy (Zhu et al. 2023). Lifestyle interventions include dietary adjustments (low-calorie diet), exercise, and weight loss. Some drugs have shown potential efficacy in clinical trials, including insulin sensitizers like pioglitazone, antioxidants such as vitamin E, and bile acid receptor agonists like obeticholic acid (Neuschwander-Tetri 2020). However, no specific pharmacological treatment for NASH has yet gained widespread recognition. Therefore, there is an urgent need to develop drugs that can specifically target NASH (Zhu et al. 2023).

Understanding the mechanisms underlying the development and progression of non-alcoholic steatohepatitis (NASH) is crucial for developing targeted therapies aimed at halting or reversing the disease’s advancement (Musso et al. 2016). The pathological mechanisms of NASH development within hepatocytes involve several stages, primarily including: Hepatic Steatosis, This stage is typically caused by an imbalance between lipid uptake, synthesis, oxidation, and transport within hepatocytes, leading to abnormal lipid accumulation (Ziolkowska et al. 2021). Oxidative Stress, The excess fatty acids within hepatocytes undergo oxidative metabolism, producing reactive oxygen species (ROS) (Besse-Patin et al. 2017). ROS induce oxidative stress, resulting in lipotoxicity that further damages cellular structures, including proteins, lipids, and DNA (Chen et al. 2020; Bathish et al. 2022; Zhu et al. 2024). Inflammatory Response, Persistent oxidative stress and lipotoxicity trigger a series of inflammatory responses. These responses activate signaling pathways such as NF-κB and stimulate M1 macrophages to release pro-inflammatory cytokines. Hepatocyte Injury and Apoptosis, Continuous oxidative stress and inflammation lead to cellular damage and death (Albhaisi and Noureddin 2021; Liu et al. 2019). Damaged hepatocytes release damage-associated molecular patterns (DAMPs), which exacerbate the inflammatory response and recruit additional immune cells to the liver. End-stage Liver Disease (Andreadou et al. 2021), Ongoing inflammation and hepatocyte injury stimulate the activation of hepatic stellate cells (HSCs), ultimately leading to liver fibrosis, cirrhosis, and hepatocellular carcinoma. Current research on specific therapeutic drugs for NASH focuses on these potential treatment targets (Musso et al. 2016).

In recent years, dental-derived mesenchymal stem cells (DMSCs) have garnered extensive attention in the field of regenerative medicine. They possess multiple advantages, such as abundant sources, easy and non-invasive acquisition, robust multi-lineage differentiation potential, and efficient tissue repair capabilities (Zhou et al. 2019). SCAPs are a type of DMSC that have been shown to promote angiogenesis and possess anti-inflammatory and tissue repair properties (Jing et al. 2022; Bakopoulou et al. 2015). In this study, we isolated apical papilla from the teeth of adolescents with indications for tooth extraction and cultured them in vitro. Exosomes were subsequently isolated from the conditioned medium. Both in vivo and in vitro NASH models were established, and the therapeutic effects of SCAP-derived exosomes were evaluated via tail vein injection and exosome-cell co-incubation. Our findings demonstrated the beneficial effects of SCAP-derived exosomes in NASH models. The results suggest that exosomes derived from apical papilla stem cells may represent a potential therapeutic agent for the treatment of NASH. These findings highlight the promising therapeutic potential of SCAP-derived exosomes in the treatment of NASH, warranting further investigation and development.

HFD is a commonly used method to induce NASH in mouse models. This model can replicate many of the metabolic and pathological characteristics of human NASH (Carreres et al. 2021; Mouskeftara et al. 2024). HFD can induce hepatic steatosis and inflammatory responses in mice within a short period (Kang et al. 2022). Due to the high-calorie intake, mice exhibit significant weight gain, liver fat accumulation, liver enlargement and pallor, and liver damage, ultimately leading to systemic impairment. In this experiment, after 12 weeks of HFD induction, mice showed significant liver and systemic damage. However, after 6 weeks of exosomes tail vein injection, the symptoms of obesity, hyperglycemia, and liver enlargement and pallor in HFD-fed mice were alleviated, and the levels of ALT and AST, indicators of liver damage, were significantly reduced. These data suggest that exosomes derived from apical papilla stem cells may help ameliorate liver and systemic damage induced by HFD in mice.

Treating HepG2 cells with OA and PA is a commonly used method to construct an in vitro model of NASH (Kang et al. 2022). This method can simulate the characteristics of NASH in vitro, including lipid accumulation, inflammatory response, and cellular injury. After constructing the in vitro NASH cell model and co-incubating with exosomes, Nile Red and Oil Red O staining revealed that lipid levels within the cells significantly decreased following exosomes co-incubation. Additionally, ROS probe staining indicated that exosomes could inhibit intracellular ROS production. These findings suggest that exosomes promote fatty acid metabolism and inhibit hepatocyte injury in vitro.

Disruption of fatty acid metabolism within hepatocytes is a critical factor in the development and progression of NASH. Therefore, promoting the balance among lipid uptake, synthesis, oxidation, and transport in hepatocytes is crucial for inhibiting the progression of NASH. PPARα is a nuclear receptor transcription factor that is highly expressed in tissues such as the liver, heart, kidneys and muscles (Boeckmans et al. 2019; Chougule et al. 2023). PPARα plays a pivotal role in fatty acid metabolism by regulating fatty acid uptake, β-oxidation, and ketogenesis (Besse-Patin et al. 2017; Jain et al. 2018). Upon binding with its ligand, PPARα undergoes a conformational change, forming a heterodimer with retinoid X receptor (RXR). This complex binds to peroxisome proliferator response elements (PPRE) in the promoter regions of target genes, initiating their transcription (Pawlak et al. 2015). Following PPARα activation, its downstream target genes, such as CPT1α and ACOX, are upregulated, facilitating mitochondrial β-oxidation of fatty acids. Additionally, PPARα activation can upregulate the expression of FABP5, enhancing intracellular fatty acid transport. PPARα activation also inhibits the expression of SREBP1c. Currently, various PPAR agonists are being investigated as potential therapeutic agents for targeting NASH.

AMPK is an intracellular energy sensor that plays a critical role in maintaining energy homeostasis (Trefts and Shaw 2021; Alghamdi et al. 2020; Wang et al. 2023a, b, c). AMPK is generally activated under conditions of low cellular energy, promoting energy balance by phosphorylating its downstream proteins. It also regulates fatty acid metabolism and is of significant importance in metabolic diseases (Trefts and Shaw 2021; Alghamdi et al. 2020). AMPK can directly phosphorylate and inhibit ACC, reducing the conversion of acetyl-CoA to malonyl-CoA, thereby activating the expression of CPT1α to reduce fatty acid synthesis and promote mitochondrial β-oxidation. Additionally, AMPK can indirectly inhibit the expression of FASN by regulating the activity of SREBP1c, thereby decreasing fatty acid synthesis.

In this experiment, we investigated the effects of exosomes on lipid metabolism genes both in vivo and in vitro. The results demonstrated that exosomes activated the phosphorylation of PPARα and AMPK, regulating the expression of the aforementioned genes. These include lipogenic genes such as SREBP1c, FASN, and ACC; lipid transport genes such as FABP5; and lipid oxidation genes such as CPT1α and Acox.

Hepatic macrophages are immune cells in the liver that undergo polarization in response to different microenvironmental signals, ultimately exhibiting distinct functional states, primarily classified into M1 and M2 macrophages (Sun and Matsukawa 2024; Vonderlin et al. 2023). M1 macrophages possess strong bactericidal and antiviral capabilities, secreting large amounts of pro-inflammatory cytokines that promote inflammatory responses. In contrast, M2 macrophages secrete anti-inflammatory cytokines such as IL10 and Arg1. CD206 is a characteristic protein on the surface of M2 macrophages (Barreby et al. 2022; Ni et al. 2024; Wang et al. 2023a, b, c). After 12 weeks of HFD induction, HFD-fed mice exhibited significant inflammatory responses, with markedly elevated serum levels of inflammatory cytokines (TNFα, IL6) and transcription levels of inflammatory cytokines (TNFα, IL6, IL1β). However, treatment with exosomes reversed this upregulation. Additionally, following exosomes treatment, the transcription levels of M2 macrophage markers Arg1, IL10, and CD206 were significantly increased, indicating a potential polarization of macrophages towards the M2 phenotype. This process may be related to the NF-kB signaling pathway, as the phosphorylation of NF-kB protein promotes the production of inflammatory cytokines such as TNFα, IL6, and IL1β. Western blot results showed that the phosphorylation levels of NF-kB were reduced after exosomes intervention. The above findings are consistent with the results of our previous research (Nie et al. 2024).

## Conclusion

This study proposes the potential of exosomes from SCAPs for the treatment of NASH and validates their anti-inflammatory and fatty acid metabolism regulatory effects. However, there are still some limitations. Firstly, the specific components within the exosomes responsible for the therapeutic effects were not further investigated. Secondly, only two treatment concentrations were selected for this study. Although the results indicated that the higher concentration had a better therapeutic effect, a broader range of concentrations and injection frequencies were not explored.

## Supplementary Information

Additional file 1.

## Data Availability

The datasets used and/or analysed during the current study are available from the corresponding author on reasonable request.

## Abbreviations

SCAPs: Stem cells from the apical papilla
MSCs: Mesenchymal stem cells
HFD: High fat diet
NASH: Non-alcoholic steatohepatitis
NAFLD: Non-alcoholic fatty liver disease
NF-κB: Nuclear factor kappa-B
AMPK: AMP-activated protein kinase
PPARα: Peroxisome proliferator-activated receptor alpha
CPT1α: Carnitine palmitoyltransferase1α
ACOX: Acyl-CoA oxidase
SREBP1c: Sterol regulatory element-binding protein 1c
ACC/ACACA: Acetyl-CoA carboxylase
FASN: Fatty acid synthase
SCD1: Stearoyl-CoA desaturase 1
IL-1β: Interleukin-1 beta
FABP5: Fatty acid-binding protein 5
AST: Aspartate aminotransferase
ALT: Alanine aminotransferase
TNFα: Tumor necrosis factor-α
IL-6: Interleukin-6
IL-1β: Interleukin-1 beta
Arg-1: Arginase-1

## References

[CR1] Albhaisi S, Noureddin M. Current and potential therapies targeting inflammation in NASH. Front Endocrinol. 2021;12: 767314. 10.3389/fendo.2021.767314. 10.3389/fendo.2021.767314 PMC8678040 34925237

[CR2] Alghamdi F, Alshuweishi Y, Salt IP. Regulation of nutrient uptake by AMP-activated protein kinase. Cell Signal. 2020;76: 109807. 10.1016/j.cellsig.2020.109807. 33038517 10.1016/j.cellsig.2020.109807

[CR3] Andreadou I, Daiber A, Baxter GF, Brizzi MF, Di Lisa F, Kaludercic N, Lazou A, Varga ZV, Zuurbier CJ, Schulz R, Ferdinandy P. Influence of cardiometabolic comorbidities on myocardial function, infarction, and cardioprotection: role of cardiac redox signaling. Free Radical Biol Med. 2021;166:33–52. 10.1016/j.freeradbiomed.2021.02.012. 33588049 10.1016/j.freeradbiomed.2021.02.012

[CR4] Bakopoulou A, Kritis A, Andreadis D, Papachristou E, Leyhausen G, Koidis P, Geurtsen W, Tsiftsoglou A. Angiogenic potential and secretome of human apical papilla mesenchymal stem cells in various stress microenvironments. Stem Cells Dev. 2015;24(21):2496–512. 10.1089/scd.2015.0197. 26203919 10.1089/scd.2015.0197 PMC4620528

[CR5] Barreby E, Chen P, Aouadi M. Macrophage functional diversity in NAFLD—more than inflammation. Nat Rev Endocrinol. 2022;18(8):461–72. 10.1038/s41574-022-00675-6. 35534573 10.1038/s41574-022-00675-6

[CR6] Bathish B, Robertson H, Dillon JF, Dinkova-Kostova AT, Hayes JD. Nonalcoholic steatohepatitis and mechanisms by which it is ameliorated by activation of the CNC-bZIP transcription factor Nrf2. Free Radical Biol Med. 2022;188:221–61. 10.1016/j.freeradbiomed.2022.06.226. 35728768 10.1016/j.freeradbiomed.2022.06.226

[CR7] Besse-Patin A, Léveillé M, Oropeza D, Nguyen BN, Prat A, Estall JL. Estrogen signals through peroxisome proliferator-activated receptor-γ coactivator 1α to reduce oxidative damage associated with diet-induced fatty liver disease. Gastroenterology. 2017;152(1):243–56. 10.1053/j.gastro.2016.09.017. 27658772 10.1053/j.gastro.2016.09.017

[CR8] Boeckmans J, Natale A, Rombaut M, Buyl K, Rogiers V, De Kock J, Vanhaecke T, Rodrigues RM. Anti-NASH drug development hitches a lift on PPAR agonism. Cells. 2019;9(1):37. 10.3390/cells9010037. 31877771 10.3390/cells9010037 PMC7016963

[CR9] Bruno S, Pasquino C, Herrera Sanchez MB, Tapparo M, Figliolini F, Grange C, Chiabotto G, Cedrino M, Deregibus MC, Tetta C, Camussi G. HLSC-derived extracellular vesicles attenuate liver fibrosis and inflammation in a murine model of non-alcoholic steatohepatitis. Mol Ther. 2020;28(2):479–89. 10.1016/j.ymthe.2019.10.016. 31757759 10.1016/j.ymthe.2019.10.016 PMC7001005

[CR10] Carreres L, Jílková ZM, Vial G, Marche PN, Decaens T, Lerat H. Modeling diet-induced NAFLD and NASH in rats: a comprehensive review. Biomedicines. 2021;9(4):378. 10.3390/biomedicines9040378. 33918467 10.3390/biomedicines9040378 PMC8067264

[CR11] Chalasani N, Younossi Z, Lavine JE, Charlton M, Cusi K, Rinella M, Harrison SA, Brunt EM, Sanyal AJ. The diagnosis and management of nonalcoholic fatty liver disease: practice guidance from the American Association for the Study of Liver Diseases. Hepatology. 2018;67(1):328–57. 10.1002/hep.29367. 28714183 10.1002/hep.29367

[CR12] Chen Z, Tian R, She Z, Cai J, Li H. Role of oxidative stress in the pathogenesis of nonalcoholic fatty liver disease. Free Radical Biol Med. 2020;152:116–41. 10.1016/j.freeradbiomed.2020.02.025. 32156524 10.1016/j.freeradbiomed.2020.02.025

[CR13] Chougule A, Baroi S, Czernik PJ, Crowe E, Chang MR, Griffin PR, Lecka-Czernik B. Osteocytes contribute via nuclear receptor PPAR-alpha to maintenance of bone and systemic energy metabolism. Front Endocrinol. 2023;14:1145467. 10.3389/fendo.2023.1145467. 10.3389/fendo.2023.1145467 PMC10173151 37181042

[CR14] Diniz TA, de Lima Junior EA, Teixeira AA, Biondo LA, da Rocha LAF, Valadão IC, Silveira LS, Cabral-Santos C, de Souza CO, Rosa Neto JC. Aerobic training improves NAFLD markers and insulin resistance through AMPK-PPAR-α signaling in obese mice. Life Sci. 2021;266: 118868. 10.1016/j.lfs.2020.118868. 33310034 10.1016/j.lfs.2020.118868

[CR15] El-Derany MO, AbdelHamid SG. Upregulation of miR-96-5p by bone marrow mesenchymal stem cells and their exosomes alleviate non-alcoholic steatohepatitis: emphasis on caspase-2 signaling inhibition. Biochem Pharmacol. 2021;190: 114624. 10.1016/j.bcp.2021.114624. 34052187 10.1016/j.bcp.2021.114624

[CR16] Ferguson D, Finck BN. Emerging therapeutic approaches for the treatment of NAFLD and type 2 diabetes mellitus. Nat Rev Endocrinol. 2021;17(8):484–95. 10.1038/s41574-021-00507-z. 34131333 10.1038/s41574-021-00507-z PMC8570106

[CR17] Friedman SL, Neuschwander-Tetri BA, Rinella M, Sanyal AJ. Mechanisms of NAFLD development and therapeutic strategies. Nat Med. 2018;24(7):908–22. 10.1038/s41591-018-0104-9. 29967350 10.1038/s41591-018-0104-9 PMC6553468

[CR18] Garcia D, Shaw RJ. AMPK: mechanisms of cellular energy sensing and restoration of metabolic balance. Mol Cell. 2017;66(6):789–800. 10.1016/j.molcel.2017.05.032. 28622524 10.1016/j.molcel.2017.05.032 PMC5553560

[CR19] Gjorgjieva M, Sobolewski C, Dolicka D, Correia de Sousa M, Foti M. miRNAs and NAFLD: from pathophysiology to therapy. Gut. 2019;68(11):2065–79. 10.1136/gutjnl-2018-318146. 31300518 10.1136/gutjnl-2018-318146

[CR20] Goonetilleke M, Kuk N, Correia J, Hodge A, Moore G, Gantier MP, Yeoh G, Sievert W, Lim R. Addressing the liver progenitor cell response and hepatic oxidative stress in experimental non-alcoholic fatty liver disease/non-alcoholic steatohepatitis using amniotic epithelial cells. Stem Cell Res Ther. 2021;12(1):429. 10.1186/s13287-021-02476-6. 34321089 10.1186/s13287-021-02476-6 PMC8317377

[CR21] Guillot A, Tacke F. Liver macrophages: old dogmas and new insights. Hepatology Communications. 2019;3(6):730–43. 10.1002/hep4.1356. 31168508 10.1002/hep4.1356 PMC6545867

[CR22] Herzig S, Shaw RJ. AMPK: guardian of metabolism and mitochondrial homeostasis. Nat Rev Mol Cell Biol. 2018;19(2):121–35. 10.1038/nrm.2017.95. 28974774 10.1038/nrm.2017.95 PMC5780224

[CR23] Hilkens P, Bronckaers A, Ratajczak J, Gervois P, Wolfs E, Lambrichts I. The angiogenic potential of DPSCs and SCAPs in an in vivo model of dental pulp regeneration. Stem Cells Int. 2017;2017:2582080. 10.1155/2017/2582080. 29018483 10.1155/2017/2582080 PMC5605798

[CR24] Jain MR, Giri SR, Bhoi B, Trivedi C, Rath A, Rathod R, Ranvir R, Kadam S, Patel H, Swain P, Roy SS, Das N, Karmakar E, Wahli W, Patel PR. Dual PPARα/γ agonist saroglitazar improves liver histopathology and biochemistry in experimental NASH models. Liver Int. 2018;38(6):1084–94. 10.1111/liv.13634. 29164820 10.1111/liv.13634 PMC6001453

[CR25] Jing X, Wang S, Tang H, Li D, Zhou F, Xin L, He Q, Hu S, Zhang T, Chen T, Song J. Dynamically bioresponsive DNA hydrogel incorporated with dual-functional stem cells from apical papilla-derived exosomes promotes diabetic bone regeneration. ACS Appl Mater Interfaces. 2022;14(14):16082–99. 10.1021/acsami.2c02278. 35344325 10.1021/acsami.2c02278

[CR26] Kalluri R, LeBleu VS. The biology, function, and biomedical applications of exosomes. Science. 2020;367(6478):eaau6977. 10.1126/science.aau6977. 32029601 10.1126/science.aau6977 PMC7717626

[CR27] Kang Y, Song Y, Luo Y, Song J, Li C, Yang S, Guo J, Yu J, Zhang X. Exosomes derived from human umbilical cord mesenchymal stem cells ameliorate experimental non-alcoholic steatohepatitis via Nrf2/NQO-1 pathway. Free Radical Biol Med. 2022;192:25–36. 10.1016/j.freeradbiomed.2022.08.037. 36096356 10.1016/j.freeradbiomed.2022.08.037

[CR28] Liu B, Deng X, Jiang Q, Li G, Zhang J, Zhang N, Xin S, Xu K. Scoparone alleviates inflammation, apoptosis and fibrosis of non-alcoholic steatohepatitis by suppressing the TLR4/NF-κB signaling pathway in mice. Int Immunopharmacol. 2019;75: 105797. 10.1016/j.intimp.2019.105797. 31421545 10.1016/j.intimp.2019.105797

[CR29] López M, Fernández-Real JM, Tomarev SI. Obesity wars: may the smell be with you. Am J Physiol Endocrinol Metab. 2023;324(6):E569–76. 10.1152/ajpendo.00040.2023. 37166265 10.1152/ajpendo.00040.2023 PMC10259866

[CR30] Meeks KAC, Bentley AR, Adeyemo AA, Rotimi CN. Evolutionary forces in diabetes and hypertension pathogenesis in Africans. Hum Mol Genet. 2021;30(R1):R110–8. 10.1093/hmg/ddaa238. 33734377 10.1093/hmg/ddaa238 PMC8117438

[CR31] Montagner A, Polizzi A, Fouché E, Ducheix S, Lippi Y, Lasserre F, Barquissau V, Régnier M, Lukowicz C, Benhamed F, Iroz A, Bertrand-Michel J, Al Saati T, Cano P, Mselli-Lakhal L, Mithieux G, Rajas F, Lagarrigue S, Pineau T, Loiseau N, Postic C, Langin D, Wahli W, Guillou H. Liver PPARα is crucial for whole-body fatty acid homeostasis and is protective against NAFLD. Gut. 2016;65(7):1202–14. 10.1136/gutjnl-2015-310798. 26838599 10.1136/gutjnl-2015-310798 PMC4941147

[CR32] Mouskeftara T, Deda O, Papadopoulos G, Chatzigeorgiou A, Gika H. Lipidomic analysis of liver and adipose tissue in a high-fat diet-induced non-alcoholic fatty liver disease mice model reveals alterations in lipid metabolism by weight loss and aerobic exercise. Molecules. 2024;29(7):1494. 10.3390/molecules29071494. 38611773 10.3390/molecules29071494 PMC11013466

[CR33] Musso G, Cassader M, Gambino R. Non-alcoholic steatohepatitis: emerging molecular targets and therapeutic strategies. Nat Rev Drug Discovery. 2016;15(4):249–74. 10.1038/nrd.2015.3. 26794269 10.1038/nrd.2015.3

[CR34] Neuschwander-Tetri BA. Therapeutic landscape for NAFLD in 2020. Gastroenterology. 2020;158(7):1984-1998.e3. 10.1053/j.gastro.2020.01.051. 32061596 10.1053/j.gastro.2020.01.051

[CR35] Ni L, Chen D, Zhao Y, Ye R, Fang P. Unveiling the flames: macrophage pyroptosis and its crucial role in liver diseases. Front Immunol. 2024;15:1338125. 10.3389/fimmu.2024.1338125. 38380334 10.3389/fimmu.2024.1338125 PMC10877142

[CR36] Nie YF, Shang JM, Liu DQ, Meng WQ, Ren HP, Li CH, Wang ZF, Lan J. Apical papilla stem cell-derived exosomes regulate lipid metabolism and alleviate inflammation in the MCD-induced mouse NASH model. Biochem Pharmacol. 2024;222: 116073. 10.1016/j.bcp.2024.116073. 38395263 10.1016/j.bcp.2024.116073

[CR37] Ohara M, Ohnishi S, Hosono H, Yamamoto K, Yuyama K, Nakamura H, Fu Q, Maehara O, Suda G, Sakamoto N. Extracellular vesicles from amnion-derived mesenchymal stem cells ameliorate hepatic inflammation and fibrosis in rats. Stem Cells Int. 2018;2018:3212643. 10.1155/2018/3212643. 30675167 10.1155/2018/3212643 PMC6323530

[CR38] Paternostro R, Trauner M. Current treatment of non-alcoholic fatty liver disease. J Intern Med. 2022;292(2):190–204. 10.1111/joim.13531. 35796150 10.1111/joim.13531 PMC9546342

[CR39] Pawlak M, Lefebvre P, Staels B. Molecular mechanism of PPARα action and its impact on lipid metabolism, inflammation and fibrosis in non-alcoholic fatty liver disease. J Hepatol. 2015;62(3):720–33. 10.1016/j.jhep.2014.10.039. 25450203 10.1016/j.jhep.2014.10.039

[CR40] Sasaki Y, Raza-Iqbal S, Tanaka T, Murakami K, Anai M, Osawa T, Matsumura Y, Sakai J, Kodama T. Gene Expression Profiles Induced by a Novel Selective Peroxisome proliferator-activated receptor α modulator (SPPARMα) pemafibrate. Int J Mol Sci. 2019;20(22):5682. 10.3390/ijms20225682. 31766193 10.3390/ijms20225682 PMC6888257

[CR41] Shi Y, Yang X, Wang S, Wu Y, Zheng L, Tang Y, Gao Y, Niu J. Human umbilical cord mesenchymal stromal cell-derived exosomes protect against MCD-induced NASH in a mouse model. Stem Cell Res Ther. 2022;13(1):517. 10.1186/s13287-022-03201-7. 36371344 10.1186/s13287-022-03201-7 PMC9652856

[CR42] Simon TG, Roelstraete B, Hartjes K, Shah U, Khalili H, Arnell H, Ludvigsson JF. Non-alcoholic fatty liver disease in children and young adults is associated with increased long-term mortality. J Hepatol. 2021;75(5):1034–41. 10.1016/j.jhep.2021.06.034. 34224779 10.1016/j.jhep.2021.06.034 PMC8530955

[CR43] Smith BK, Marcinko K, Desjardins EM, Lally JS, Ford RJ, Steinberg GR. Treatment of nonalcoholic fatty liver disease: role of AMPK. Am J Physiol Endocrinol Metab. 2016;311(4):E730–40. 10.1152/ajpendo.00225.2016. 27577854 10.1152/ajpendo.00225.2016

[CR44] Sumida Y, Yoneda M. Current and future pharmacological therapies for NAFLD/NASH. J Gastroenterol. 2018;53(3):362–76. 10.1007/s00535-017-1415-1. 29247356 10.1007/s00535-017-1415-1 PMC5847174

[CR45] Sun C, Matsukawa A. Role of macrophages in liver fibrosis. Acta Med Okayama. 2024;78(1):1–8. 10.18926/AMO/66664. 38419308 10.18926/AMO/66664

[CR46] Trefts E, Shaw RJ. AMPK: restoring metabolic homeostasis over space and time. Mol Cell. 2021;81(18):3677–90. 10.1016/j.molcel.2021.08.015. 34547233 10.1016/j.molcel.2021.08.015 PMC8549486

[CR47] Vonderlin J, Chavakis T, Sieweke M, Tacke F. The multifaceted roles of macrophages in NAFLD pathogenesis. Cell Mol Gastroenterol Hepatol. 2023;15(6):1311–24. 10.1016/j.jcmgh.2023.03.002. 36907380 10.1016/j.jcmgh.2023.03.002 PMC10148157

[CR48] Wang C, Zhou H, Wu R, Guo Y, Gong L, Fu K, Ma C, Peng C, Li Y. Mesenchymal stem cell-derived exosomes and non-coding RNAs: regulatory and therapeutic role in liver diseases. Biomed Pharmacother. 2023a;157: 114040. 10.1016/j.biopha.2022.114040. 36423545 10.1016/j.biopha.2022.114040

[CR49] Wang X, Tan X, Zhang J, Wu J, Shi H. The emerging roles of MAPK-AMPK in ferroptosis regulatory network. Cell Communication and Signaling. 2023b;21(1):200. 10.1186/s12964-023-01170-9. 37580745 10.1186/s12964-023-01170-9 PMC10424420

[CR50] Wang L, Yan Y, Wu L, Peng J. Natural products in non-alcoholic fatty liver disease (NAFLD): novel lead discovery for drug development. Pharmacol Res. 2023c;196: 106925. 10.1016/j.phrs.2023.106925. 37714392 10.1016/j.phrs.2023.106925

[CR51] Xu L, Zhang W, Kwak KJ. Extracellular vesicles and their roles in the pathogenesis of chronic liver diseases. J Hepatol. 2017;67(2):442–53. 10.1016/j.jhep.2017.04.018.

[CR52] Younossi Z, Anstee QM, Marietti M, Hardy T, Henry L, Eslam M, George J, Bugianesi E. Global burden of NAFLD and NASH: trends, predictions, risk factors and prevention. Nat Rev Gastroenterol Hepatol. 2018;15(1):11–20. 10.1038/nrgastro.2017.109. 28930295 10.1038/nrgastro.2017.109

[CR53] Zhai Q, Dong Z, Wang W, Li B, Jin Y. Dental stem cell and dental tissue regeneration. Frontiers of Medicine. 2019;13(2):152–9. 10.1007/s11684-018-0628-x. 29971640 10.1007/s11684-018-0628-x

[CR54] Zhang W, Lang R. Macrophage metabolism in nonalcoholic fatty liver disease. Front Immunol. 2023;14:1257596. 10.3389/fimmu.2023.1257596. 37868954 10.3389/fimmu.2023.1257596 PMC10586316

[CR55] Zhao Z, Zhang L, Ocansey DKW, Wang B, Mao F. The role of mesenchymal stem cell-derived exosome in epigenetic modifications in inflammatory diseases. Front Immunol. 2023;14:1166536. 10.3389/fimmu.2023.1166536. 37261347 10.3389/fimmu.2023.1166536 PMC10227589

[CR56] Zhong H, Dong J, Zhu L, Mao J, Dong J, Zhao Y, Zou Y, Guo M, Ding G. Non-alcoholic fatty liver disease: pathogenesis and models. Am J Transl Res. 2024;16(2):387–99. 10.62347/KMSA5983. 38463579 10.62347/KMSA5983 PMC10918142

[CR57] Zhou T, Pan J, Wu P, Huang R, Du W, Zhou Y, Wan M, Fan Y, Xu X, Zhou X, Zheng L, Zhou X. Dental follicle cells: roles in development and beyond. Stem Cells Int. 2019;2019:9159605. 10.1155/2019/9159605. 31636679 10.1155/2019/9159605 PMC6766151

[CR58] Zhou J, Tripathi M, Ho JP, Widjaja AA, Shekeran SG, Camat MD, James A, Wu Y, Ching J, Kovalik JP, Lim KH, Cook SA, Bay BH, Singh BK, Yen PM. Thyroid hormone decreases hepatic steatosis, inflammation, and fibrosis in a dietary mouse model of nonalcoholic steatohepatitis. Thyroid. 2022a;32(6):725–38. 10.1089/thy.2021.0621. 35317606 10.1089/thy.2021.0621

[CR59] Zhou S, You H, Qiu S, Yu D, Bai Y, He J, Cao H, Che Q, Guo J, Su Z. A new perspective on NAFLD: focusing on the crosstalk between peroxisome proliferator-activated receptor alpha (PPARα) and farnesoid X receptor (FXR). Biomed Pharmacother. 2022b;154: 113577. 10.1016/j.biopha.2022.113577. 35988420 10.1016/j.biopha.2022.113577

[CR60] Zhu L, Zhang J, Yang H, Li G, Li H, Deng Z, Zhang B. Propolis polyphenols: a review on the composition and anti-obesity mechanism of different types of propolis polyphenols. Front Nutr. 2023;10:1066789. 10.3389/fnut.2023.1066789. 37063322 10.3389/fnut.2023.1066789 PMC10102383

[CR61] Zhu Y, Tan JK, Liu J, Goon JA. Roles of traditional and next-generation probiotics on non-alcoholic fatty liver disease (NAFLD) and non-alcoholic steatohepatitis (NASH): a systematic review and network meta-analysis. Antioxidants. 2024;13(3):329. 10.3390/antiox13030329. 38539862 10.3390/antiox13030329 PMC10968178

[CR62] Ziolkowska S, Binienda A, Jabłkowski M, Szemraj J, Czarny P. The interplay between insulin resistance, inflammation, oxidative stress, base excision repair and metabolic syndrome in nonalcoholic fatty liver disease. Int J Mol Sci. 2021;22(20):11128. 10.3390/ijms222011128. 34681787 10.3390/ijms222011128 PMC8537238

[CR63] Zobeiri M, Parvizi F, Kalhori MR, Majnooni MB, Farzaei MH, Abdollahi M. Targeting miRNA by natural products: a novel therapeutic approach for nonalcoholic fatty liver. Evid Based Complement Alternat Med. 2021;2021(2021):6641031. 10.1155/2021/6641031. 34426744 10.1155/2021/6641031 PMC8380168

[CR64] Zou Y, Liao L, Dai J, Mazhar M, Yang G, Wang H, Dechsupa N, Wang L. Mesenchymal stem cell-derived extracellular vesicles/exosome: a promising therapeutic strategy for intracerebral hemorrhage. Regen Ther. 2023;22:181–90. 10.1016/j.reth.2023.01.006. 36860266 10.1016/j.reth.2023.01.006 PMC9969203

[CR65] Zuccarini M, Giuliani P, Di Liberto V, Frinchi M, Caciagli F, Caruso V, Ciccarelli R, Mudò G, Di Iorio P. Adipose stromal/stem cell-derived extracellular vesicles: potential next-generation anti-obesity agents. Int J Mol Sci. 2022;23(3):1543. 10.3390/ijms23031543. 35163472 10.3390/ijms23031543 PMC8836090

[CR66] Zuo Q, Park NH, Lee JK, Santaliz-Casiano A, Madak-Erdogan Z. Navigating nonalcoholic fatty liver disease (NAFLD): exploring the roles of estrogens, pharmacological and medical interventions, and life style. Steroids. 2024;203: 109330. 10.1016/j.steroids.2023.109330. 37923152 10.1016/j.steroids.2023.109330

