# Supplementary figures and images for: Exosomes derived from apical papilla stem cells improve NASH by regulating fatty acid metabolism and reducing inflammation
Source: Mol Med. 2024 Oct 26;30:186. doi: 10.1186/s10020-024-00945-1 (PMC11512503; doi:10.1186/s10020-024-00945-1)

Figure1

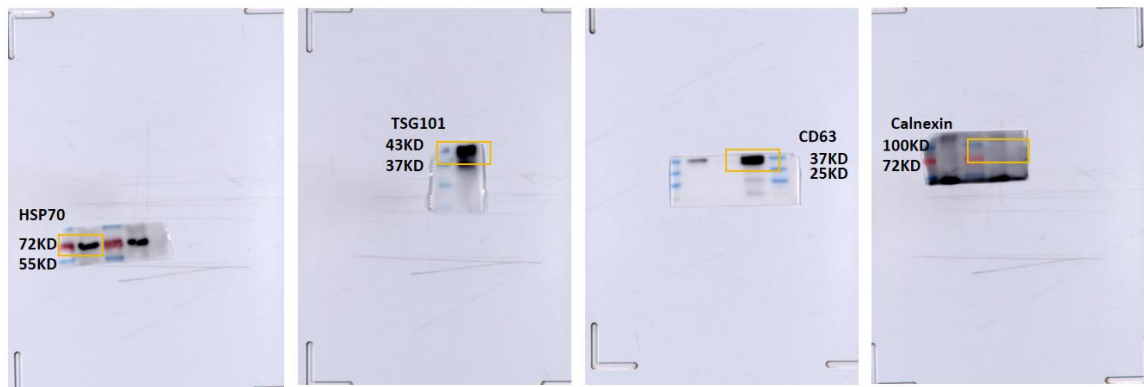

Figure4-B

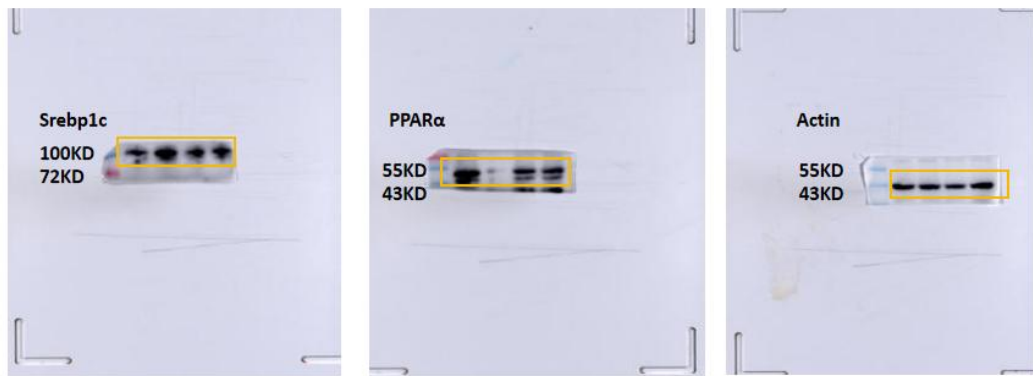

Figure4-C

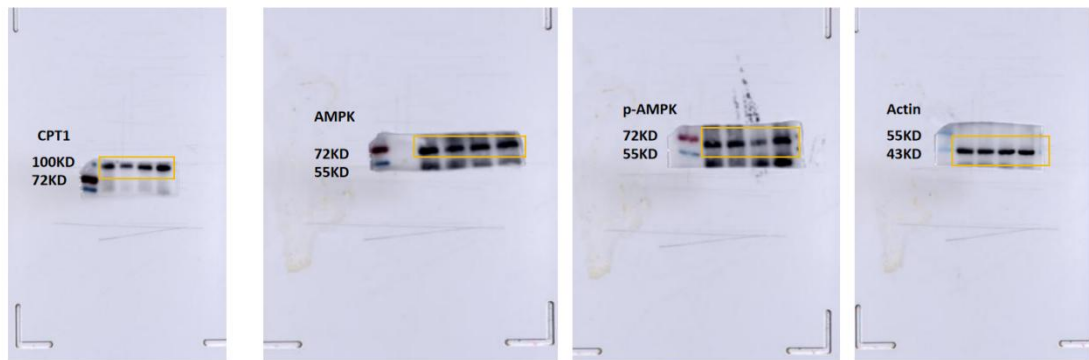

Figure5-D

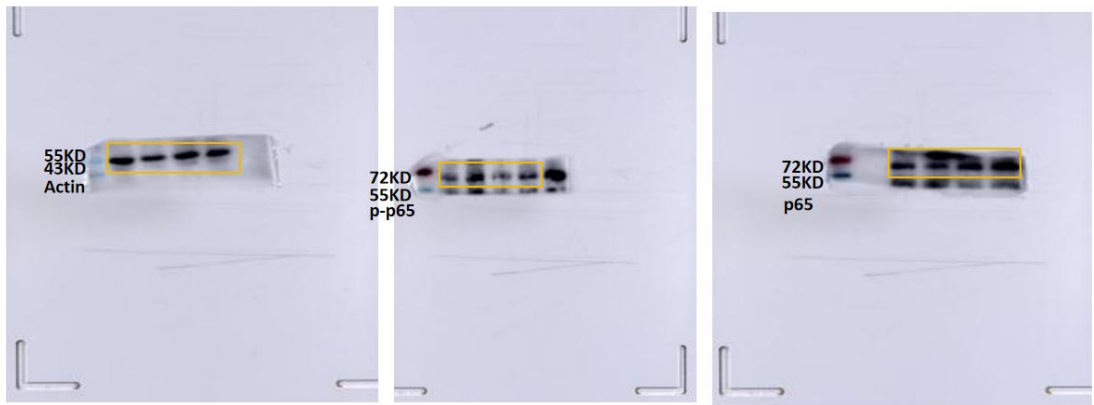

Figure6-E

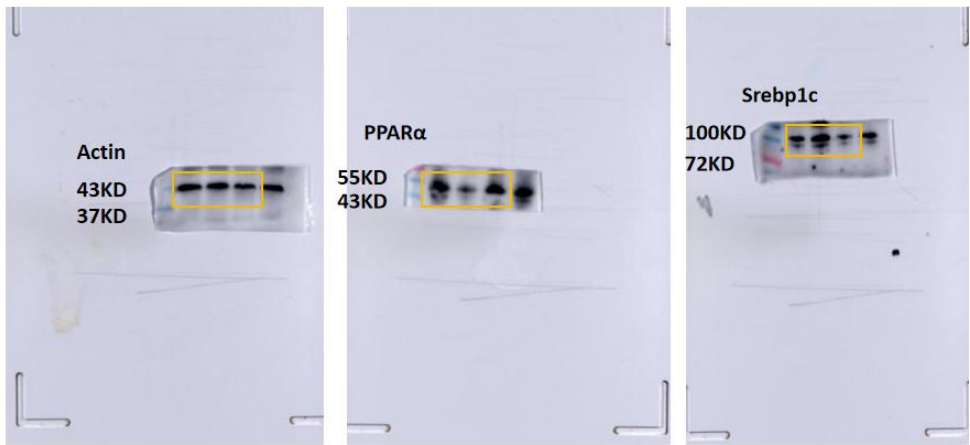

Figure6-F

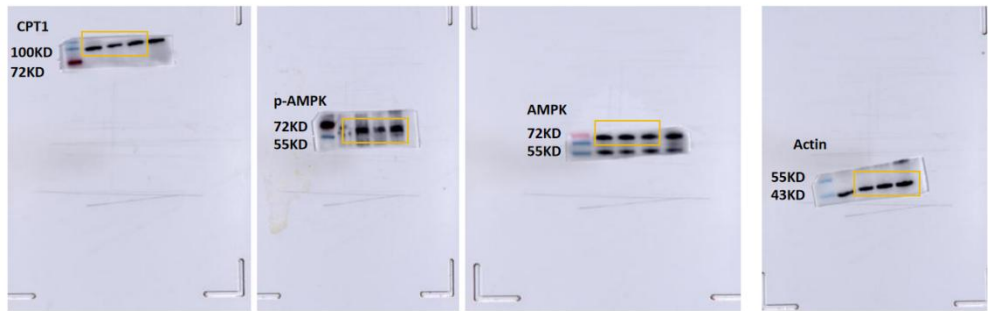

Supplement: Supplementary file 1 — Additional file 1. [file 10020_2024_945_MOESM1_ESM.pdf]
